# Protocol for efficient germination of *Castilleja* (Orobanchaceae) for chemical haustorium induction and parasite-host interaction studies

**DOI:** 10.1016/j.xpro.2025.103753

**Published:** 2025-04-08

**Authors:** Sagar Bashyal, Lena Maria Müller, Joanne Chory, Marco Bürger

**Affiliations:** 1 Plant Molecular and Cellular Biology Laboratory, Salk Institute for Biological Studies, La Jolla, CA 92037, USA; 2 School of Biological Sciences, University of California, San Diego, La Jolla, CA 92093, USA; 3 Howard Hughes Medical Institute, Salk Institute for Biological Studies, 10010 North Torrey Pines Road, La Jolla, CA 92037, USA

**Keywords:** Cell Biology, Developmental biology, Microscopy, Plant sciences, Molecular Biology, Evolutionary biology

## Abstract

We present a protocol for germinating *Castilleja* seeds and growing seedlings for hormone treatments and plant-plant interaction assays. We describe steps for seed sterilization, stratification, and transplantation, ensuring germination and seedling development. We detail procedures for haustorium formation, including strigolactone treatment and co-inoculation to induce root interactions with a host plant. This protocol includes optimized nutrient conditions and microscopy techniques to ensure experimental reproducibility. This approach enables reliable investigation of haustorium formation in *Castilleja* under laboratory conditions.

For complete details on the use and execution of this protocol, please refer to Bürger et al.^1^

## Highlights

• Protocol for efficient germination of *Castilleja* seeds under controlled conditions
• Instructions for chemical haustorium induction using strigolactone treatment
• Step-by-step procedures for establishing *Castilleja*-host interactions
• Standardized microscopy approaches for visualizing haustorium formation and development

## Abstract (editor highlights)

Publisher’s note: Undertaking any experimental protocol requires adherence to local institutional guidelines for laboratory safety and ethics.

## Graphical abstract

**Figure:**
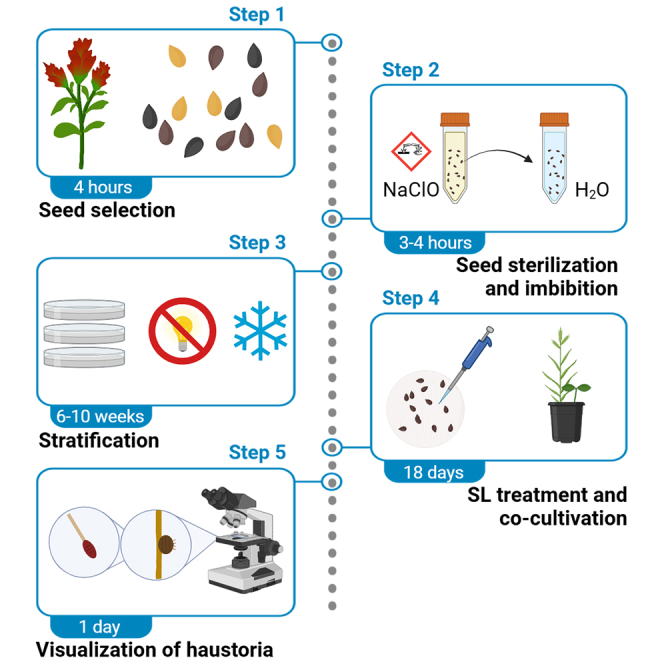

## Before you begin

*Castilleja* species, members of the Orobanchaceae family, are facultative root hemiparasites capable of obtaining nutrients from host plants through a root-like structure called haustorium.^2^^,^^3^^,^^4^^,^^5^ Despite their broad host range and ecological significance, protocols for efficiently growing *Castilleja* seedlings remain limited. In this protocol, we focused on *Castilleja affinis*, a species abundant in California’s coastal regions, particularly in chaparral and coastal scrub habitats up to 1800 meters in elevation. This species exhibits bright red to orange-red inflorescences, blooms from March to June, and serves as an excellent model for studying plant parasitism due to its widespread distribution throughout California and Baja California plant communities. While studies have established that different *Castilleja* species require prolonged moist chilling for weeks to months to break seed dormancy,^6^ detailed and stepwise protocols for efficient seed germination, seedling establishment, and successful host interactions under controlled laboratory conditions have not been established. Recently, we demonstrated that strigolactones initiate the formation of haustorium-like structures in *Castilleja*.^1^ Here, we outline an optimized approach for *Castilleja* seed selection and sterilization, seed stratification and germination, seedling growth with a host, and hormone treatment, using *C. affinis* as an example. The stepwise method provided here will enable future research on this genus. Given its facultative parasitic nature and broad host range, the genus *Castilleja* represents a valuable model for studying plant-plant interactions and can help elucidate the molecular and physiological mechanisms underlying hemiparasitism. As parasitic plants influence large plant communities and nutrient dynamics, establishing a reliable protocol for *Castilleja* cultivation is essential for ecological studies and conservation efforts. The successful parasitism of Purple False-Brome (*Brachypodium distachyon*) demonstrated in this protocol suggests potential applications in invasive species management, as native *Castilleja* could serve as a natural biocontrol agent against this introduced Mediterranean grass in California ecosystems.

### Institutional permissions

The *Castilleja* species used in this study are not listed as threatened or endangered under state or federal regulations.

Researchers must ensure compliance with institutional guidelines and obtain necessary approvals before initiating experiments with *Castilleja* species. All procedures, including seed germination, host interactions, and hormone treatments, should be conducted following institutional biosafety regulations. Additionally, researchers should be aware that several *Castilleja* species are listed as threatened or endangered under state or federal regulations. Before collecting or working with any *Castilleja* species, investigators must verify the conservation status of their target species and obtain the required permits from relevant authorities.

### Prepare solution for surface sterilization of *Castilleja* seeds

**Timing: 20 min**

1. Prepare 70% (v/v) ethanol for seeds surface sterilization.
  a. Measure 700 mL of 100% ethanol and transfer it to a 1000 mL flask.
  b. Add deionized water to bring the final volume to 1000 mL.
  c. Store in a sealed glass bottle at room temperature.
2. For an alternate seed sterilization approach, prepare 3% (v/v) NaClO (bleach) solution (to be done in a fume hood).
3. Prepare autoclaved deionized water for washing seeds.
  a. Autoclave deionized water in glass bottles at 121°C, 15 psi for 20 min.
  b. Store at room temperature until use.

### Prepare Fähraeus media plates for seed germination

**Timing: 1–2 h (stock solution preparation) + 1 h (F media preparation and sterilization) + 20–45 min (solidification of media plates)**

***Note:*** Solid Fähraeus (F) medium^7^ is prepared for *C. affinis* seed germination and seedling development, which can further be used to study plant-plant interactions. It should be used immediately after autoclaving. Alternatively, the media can be stored at 4°C for later use, requiring melting by microwaving before dispensing.

4. Prepare 800 mL of ready-to-use F-medium:
  a. Pipette the required amount for each F medium stock solution (excluding Gelzan agar) into a 1 L bottle with 750 mL of nuclease-free or deionized water. The recipe is provided in the table below.
  b. Place a magnetic stir bar inside the bottle and stir the solution on a magnetic stirrer to dissolve any precipitates.
5. Carefully adjust the pH to 7.4 using potassium hydroxide (KOH).
6. Weigh 5 g of Gelzan agar and add it to the media.
  a. Continue mixing on the magnetic stirrer until all agar is dissolved.
  b. Ensure that no Gelzan clumps remain at the bottom.
7. Bring the total volume to 800 mL with deionized water.

**CRITICAL:** The medium will not solidify properly if the pH is not maintained. Keep the pH in the 7.2–7.4 range.

8. Autoclave the medium using a liquid cycle at 121°C, 15 psi for 40 min, keeping the bottle cap slightly loose to prevent pressure buildup.
9. Prepare media plates:
  a. Allow the autoclaved F-medium to cool down to approximately 60°C–80°C.
  b. Mix the medium by slowly shaking the bottle and pour it into 12 × 12 cm square plates.
    For different plate types:
    i. Flat media plates: Pour 90 mL to fully cover the surface.
    ii. Slanted media plates: Position at ∼20° angle and pour medium into the bottom ⅔ of the plate. Keep the top portion free of medium.
  c. Leave the plates undisturbed in the laminar airflow cabinet until the medium is fully solidified.
    **CRITICAL:** Ensure that the lid is not completely closed to expose the medium to air in the laminar airflow cabinet. This helps to avoid condensation in the lid.
    ***Note:*** It is best to prepare the solid F-media plates right before use. This minimizes potential changes in media composition during storage.

### Prepare ½ Hoagland’s nutrient solution

**Timing: 1–2 h (stock solution preparation) + 30 min (dilution and pH adjustment)**

10. Pipette the required amounts for each stock solution into a glass bottle containing approximately 950 mL of deionized (DI) water. (Refer to the recipe table below).
11. Place a magnetic stir bar into the bottle.
12. Stir the solution in a magnetic stirrer until all components are fully mixed.
13. Adjust the pH to 6.1 using NaOH.
14. Bring the total volume to 1 L with deionized water.
15. Mix well to ensure homogeneity.

***Note:*** Autoclaving is not essential but recommended for long-term storage or when working under sterile conditions.

***Note:*** For large-scale experiments, preparing 10 L of ½ Hoagland’s solution in a plastic storage tank is recommended, as plants require biweekly watering with nutrient media.

### Preparation of the chemical strigolactone analog GR24 5DO

**Timing: 20 min**

16. Dissolve the GR24 5DO powder in an organic solvent, such as dimethyl sulfoxide (DMSO), methanol, or anhydrous acetone to obtain a 10 mM stock solution.

***Note:*** The molecular weight of GR24 5DO is 298.29 g/mol. Aliquot the stock solution into small volumes and store at −20°C. Aliquoting minimizes freeze-thaw cycles and maintains reagent stability over time, ensuring consistency across experiments. The stock solution can be stored for several years.

17. Transfer 1 μL of the 10 mM GR24 5DO stock solution into a fresh tube containing 10 mL of deionized H_2_O to obtain a 1 μM GR24 5DO working solution.
18. Sterile-filter the working solution.

**CRITICAL:** The working solution must be made fresh before the experiment and used promptly. Strigolactones are not very stable in aqueous solutions and undergo spontaneous hydrolysis, rendering them biologically inactive. Even when stored at 4°C, GR24 5DO shows approximately 20% degradation after 8 h in H_2_O.^8^

### Prepare sterilized tools for seed handling

**Timing: 20–30 min**

All materials and tools must be sterilized and handled under aseptic conditions to prevent contamination.

19. Wipe down the laminar airflow cabinet and all instruments with 70% ethanol before starting experiments.
20. Wipe pipettes thoroughly with 70% ethanol before handling sterile solutions.
21. Autoclave pipette tips using a gravity cycle at 121°C, 15 psi for 20 min before use.
22. Flame-sterilize tweezers before handling seeds.
23. Keep all sterilized tools inside the laminar airflow cabinet, ensuring they do not touch non-sterile surfaces.
24. Wrap 90–100 mm diameter Whatman filter paper discs in aluminum foil and autoclave using a gravity cycle at 121°C, 15 psi for 20 min. The filters should fit in standard-size petri dishes (100 × 15 mm).
25. Petri plates and square plates should be purchased sterile and only be opened inside the laminar airflow cabinet.
26. Use sterile micropore tape to seal plates after inoculation to prevent contamination.
27. Bleach and rinse cone-tainers with water before use.
28. Wash quartz sand 5–7 times with tap water to remove impurities, then autoclave using a gravity cycle at 121°C, 15 psi for 40 min.
29. Autoclave vermiculite using a gravity cycle at 121°C, 15 psi for 40 min to eliminate contaminants.

## Key resources table

**Table:** 

REAGENT or RESOURCE	SOURCE	IDENTIFIER
**Chemicals, peptides, and recombinant proteins**		

Ethanol (100%)	Koptec	64-17-5
Sodium hypochlorite (NaClO)	WAXIE	11003428432
Fähraeus (F) medium components	Sigma-Aldrich	N/A
Hoagland’s solution components	Sigma-Aldrich	N/A
Dimethyl sulfoxide	Sigma-Aldrich	317275
GR24 5DO (chemical strigolactone analog)	StrigoLab	EN1010
Deionized water	Laboratory	N/A

**Experimental models: Organisms/strains**		

*Castilleja affinis* (hemiparasitic plant)	Collected from natural populations	N/A
*Brachypodium distachyon* (host plant)	Collected from natural populations	N/A

**Other**		

Zeiss Axio Zoom.V16	Zeiss	N/A
Leica M205 FA stereomicroscope	Leica Microsystems	N/A
Keyence BZ-X810	Keyence	N/A
Whatman filter paper	Sigma-Aldrich	WHA1001824
Petri dishes (sterile)	Kord-Valmark	KORD-2900
Square plates (12 × 12 cm, sterile)	Greiner Bio-One	688102
Micropore tape	VWR	75788-400
Cone-tainers (20.5 cm)	Stuewe & Sons	SC10R
Quartz sand (play sand)	Quikrete	1113
Vermiculite	Vigoro	N/A
pH meter		

## Materials and equipment

**Table:** Stock solutions for F-medium (1 L each)

Reagent	Final concentration	Amount
CaCl_2_·2H_2_O	0.9 M	132.31 g
MgSO_4_·7H_2_O	0.5 M	123.23 g
Ferric Citrate	20.0 mM	4.90 g
NH_4_NO_3_	0.5 M	40.02 g
KH_2_PO_4_	0.35 M	47.63 g
Na_2_HPO_4_·2H_2_O	0.2 M	35.60 g
H_3_BO_3_	1.6 mM	98.9 mg
MnCl_2_·4H_2_O	262 μM	51.9 mg
CuSO_4_·5H_2_O	207 μM	51.7 mg
ZnSO_4_·7H_2_O	46 μM	13.2 mg
Na_2_MoO_4_·2H_2_O	160 μM	38.7 mg

Store at 4°C for up to 6 months.

**Table:** F-medium, pH 7.4 (800 mL)

Reagent	Final concentration	Amount
CaCl_2_·2H_2_O	0.9 mM	0.8 mL
MgSO_4_·7H_2_O	0.5 mM	0.8 mL
Ferric Citrate	20 μM	0.8 mL
NH_4_NO_3_	1 mM	1.6 mL
KH_2_PO_4_	20.3 μM	46.4 μL
Na_2_HPO_4_·2H_2_O	10 μM	40 μL
H_3_BO_3_	1.6 μM	80 μL
MnCl_2_·4H_2_O	0.262 μM	26.4 μL
CuSO_4_·5H_2_O	0.207 μM	26.4 μL
ZnSO_4_·7H_2_O	0.046 μM	26.4 μL
Na_2_MoO_4_·2H_2_O	0.160 μM	26.4 μL
MES	1.12 mM	174.4 mg
Gelzan	6.25 g/L	5.0 g
ddH_2_O	N/A	To 800 mL
Total	N/A	800 mL

Amounts are stock solutions. Use immediately after autoclaving, or store at 4°C for up to 2 weeks.

**Table:** Stock solutions for modified ½ Hoagland’s solution (1 L each)

Reagent	Final concentration	Amount
Ca(NO_3_)_2_·4H_2_O (part of Stock I)	1.0 M	236.2 g
KNO_3_ (part of Stock I)	1.0 M	101.1 g
MgSO_4_·7H_2_O (Stock II)	1.0 M	246.48 g
NaFeEDTA (Stock III)	0.1 M	36.7 g
KH_2_PO_4_ (Stock IV)	0.1 M	13.6 g
H_3_BO_3_ (part of Stock V)	10 mM	618.4 mg
Na_2_MoO_4_·2H_2_O (part of Stock V)	0.2 mM	48.4 mg
ZnSO_4_·7H_2_O (part of Stock V)	1.0 mM	287.6 mg
MnCl_2_·4H_2_O (part of Stock V)	2.0 mM	395.8 mg
CuSO_4_·5H_2_O (part of Stock V)	0.5 mM	124.8 mg
CoCl_2_·6H_2_O (part of Stock V)	0.2 mM	47.6 mg
MES (Stock VI)	0.5 mM	97.5 mg

Store at room temperature for up to 6 months.

**Table:** Modified ½ Hoagland’s solution with 20 μM P_i_, pH 6.1

Reagent	Final concentration	Amount
Stock I	Ca(NO_3_)_2_·4H_2_O: 2.5 mM, KNO_3_: 2.5 mM	2.5 mL
Stock II	MgSO_4_·7H_2_O: 1.0 mM	1.0 mL
Stock III	NaFeEDTA: 50 μM	0.5 mL
Stock IV	KH_2_PO_4_: 20 μM	0.2 mL
Stock V	H_3_BO_3_: 10 μM, Na_2_MoO_4_·2H_2_O: 0.2 μM, ZnSO_4_·7H_2_O: 1.0 μM, MnCl_2_·4H_2_O: 2.0 μM, CuSO_4_·5H_2_O: 0.5 μM, CoCl_2_·6H_2_O: 0.2 μM 1.0 mL	1.0 mL
Stock VI	MES: 0.5 μM	1.0 mL
Deionized Water	N/A	To 1 L
Total	N/A	1 L

Store at room temperature for up to 6 months.

## Step-by-step method details

### Seed selection and sterilization of *Castilleja* seeds

**Timing: 30–45 min (seed selection and sterilization) + 4 h (seed imbibition)**

***Note:*** Always wear gloves while handling seeds to maintain sterility.

1. Collect mature seed pods of *C. affinis.*

**Figure 1:**
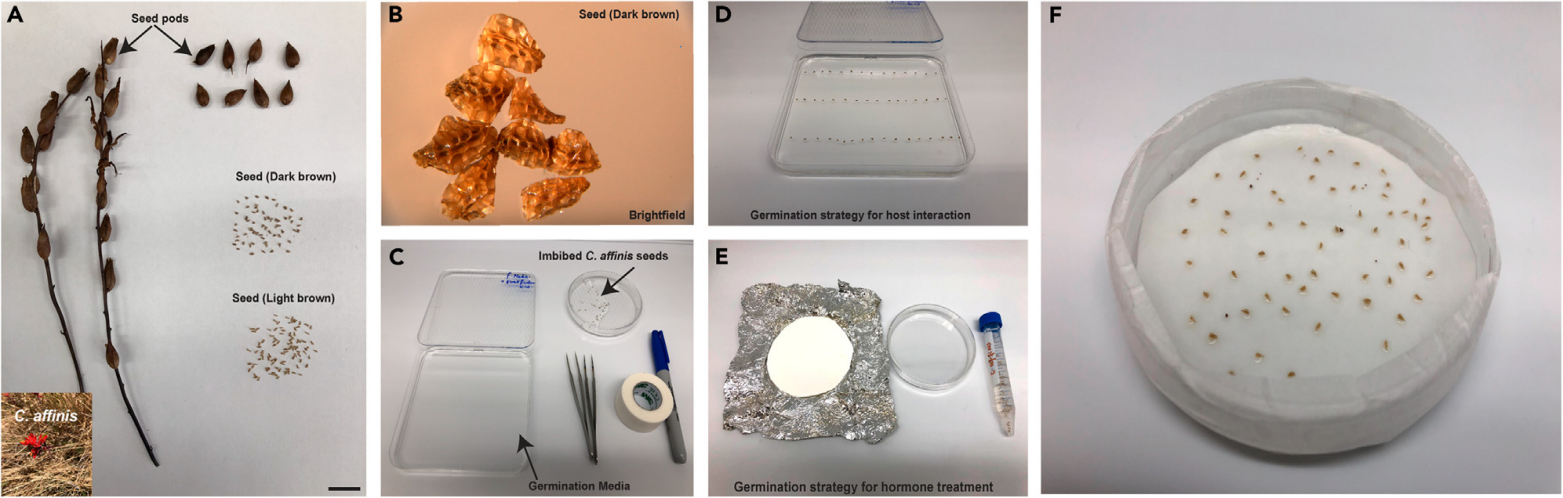
Seed selection, sterilization, and germination strategies for *Castilleja affinis* (A) Mature *C. affinis* seed pods with extracted seeds. Dark brown to blackish-brown seeds exhibit higher viability than lighter brown seeds. Scale bar: 1 cm. (B) Bright-field microscopy image of dark brown *C. affinis* seeds showing their distinct triangular shape. (C) Preparation for sowing *C. affinis* seeds on F-medium plates for use in plant-plant interaction assays. Imbibed seeds are handled using sterilized tools and placed on solidified F-medium in square plates for germination. (D) Sowing of *C. affinis* seeds on F-medium plates, arranged in rows to ensure even spacing and optimal seedling development for subsequent plant-plant interaction experiments. (E) Initial setup for hormone treatment assays. *C. affinis* seeds are placed on a sterile Whatman filter paper inside a Petri dish. (F) *C. affinis* seeds distributed on Whatman filter paper before stratification at 4°C. These seeds will later be used for hormone treatment assays.

***Note:*** Each pod contains approximately 100 triangular seeds (Figures 1A and 1B).

2. Gently crush the pods over a clean white paper sheet to release the seeds.
3. Carefully select dark brown to blackish-brown seeds, as these have a higher viability rate than lighter-colored seeds (Figure 1A).

***Note:*** This variation can likely be attributed to the after-ripening process, the need for which varies among *Castilleja* species^6^ but generally occurs in the Orobancaceae.^9^

***Note:*** Counting seeds precisely can be challenging. Use more seeds rather than too few. Any extra seeds can be propagated.

4. Handle seeds gently to avoid mechanical damage.
5. Transfer the selected seeds into a Falcon tube:
  a. Use a 15 mL or 50 mL Falcon tube to ensure free movement during seed sterilization and imbibition.

***Note:*** Avoid using 2 mL tubes as they restrict seed movement.

6. Sterilize seeds:
  a. Fill half of the Falcon tube with 70% ethanol.
  b. Surface- sterilize seeds by gently rotating the Falcon tube on lab orbital shaker for 8 min.
  c. Alternatively, use ∼3% NaClO (bleach) instead of ethanol for 7 min.

**CRITICAL:** From this step onward, the Falcon tubes containing the seeds should only be opened inside a laminar airflow cabinet to prevent contamination during stratification and germination (see troubleshooting steps below).

7. Discard the ethanol or bleach solution and rinse seeds four times with sterile autoclaved deionized water.
8. Imbibe seeds:
  a. Fill two-thirds of the Falcon tube with autoclaved deionized water.
  b. Gently agitate the tubes on a lab orbital shaker at room temperature for 4 h to allow seed imbibition.
9. After imbibition:
  a. Discard the water.
  b. Rinse the seeds twice with autoclaved deionized water.
  c. Leave a small volume of water in the tube to prevent seeds from drying out until further processing.

### Seed stratification and growth of seedlings

**Timing: 6–10 weeks (seed stratification) + 7–14 days (seedling growth)**

***Note:*** Whatman paper was used to sow and stratify seeds in strigolactone treatment assay to provide a sterile, hormone-responsive surface for direct observation of haustorium-formation upon hormone treatment within a few days, as strigolactone treatment is inefficient in media-based systems due to limited long-term stability of strigolactones in aqueous solutions. In contrast, F medium was used to germinate seedlings for plant-plant interaction assays to provide a moist environment with ample mineral nutrients for efficient germination and to support growth of seedlings with long roots and healthy shoots, which is critical for a successful plant-plant interaction assay.

**CRITICAL:** The stratification period varies due to species-specific differences within the *Castilleja* genus, experimental goals and seed batch variation.^6^ A minimum of 6 weeks of stratification is recommended for strigolactone treatment on filter paper. However, while 6 weeks is sufficient for stratification and initial root emergence also on F-medium, extending the period by an additional 2–4 weeks allows seedlings to develop cotyledons and reach a root length of 1–2 cm. These more developed seedlings are easier to transfer to new media plates prior to co-cultivation with the host plant. Seeds should be monitored regularly for germination progress (see troubleshooting steps below).

**Figure 2:**
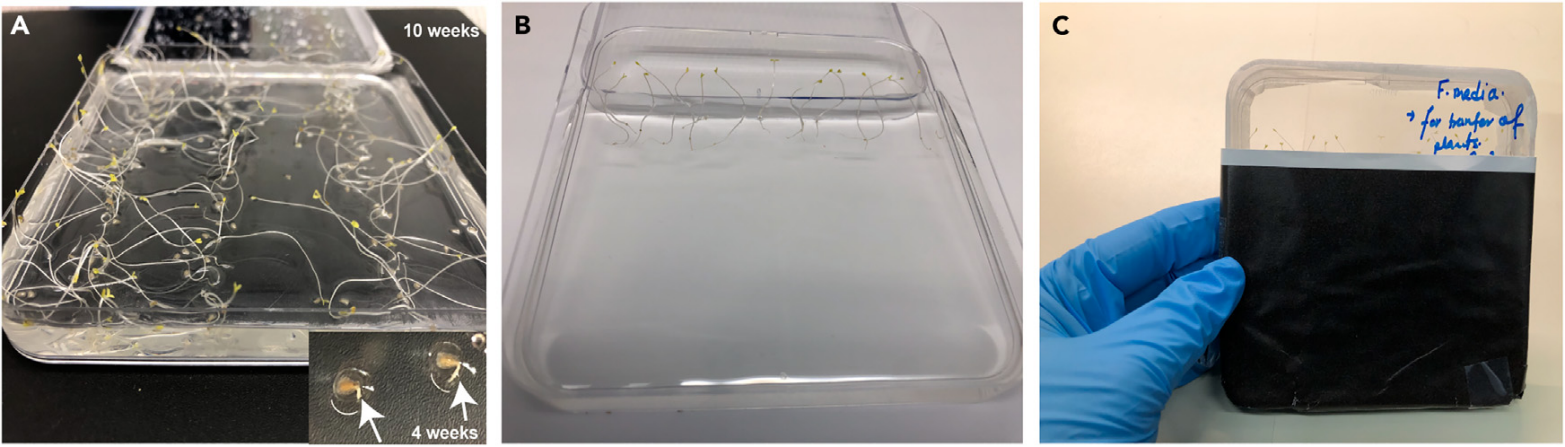
Stratification and seedling growth of *Castilleja affinis* on F-medium plates (A) *C. affinis* seedlings germinated on flat F-medium plates after 10 weeks of stratification at 4°C. The inset image shows an early germination stage of 4 weeks, where radicle emergence is visible (white arrows). (B) *C. affinis* seedlings transferred to slanted F-medium plates for continued growth, ensuring roots grow into the media while shoots develop in the empty space above. (C) Two-thirds of the plate are covered with black paper to prevent roots from direct light exposure. With this setup. seedlings are grown for 2 additional weeks in light, enhancing root elongation while allowing shoot development in the uncovered section of the plate.
  h. Transfer the seedlings to slanted F-medium plates, ensuring each seedling is spaced 5 mm apart.
  i. Position seedlings so that roots lie flat on top of the F-medium. Shoots are positioned over the portion of the plate that is free of F medium (Figure 2B).
  j. Seal the plates with micropore tape to maintain sterility.
  k. Cover the lower two-thirds of the plate with a paper cover to minimize light exposure of the roots and encourage optimal root growth (Figure 2C).
    ***Note:*** The black paper covers are handmade. Papers were printed black on both sides and cut to the shape of the square plate. Instead of using paper, aluminum foil can also be used.
  l. Maintain plates at a ∼45° angle under a grow light at room temperature for 2 weeks.
    ***Note:*** We used commercially available plant grow lights, regulated under a 16-h light/8-h dark cycle using a timer outlet.
    **CRITICAL:** For co-cultivation of *Castilleja* seedlings with host plants, *Brachypodium distachyon* seedling germination (see below) should be started once the *Castilleja* seedlings are transferred to light and room temperature (Step 25).

10. Sow *C. affinis* seeds on Whatman filter paper (for strigolactone treatment)
  a. Place sterile Whatman filter paper disks inside two Petri dishes (Figure 1E).
    ***Note:*** Use one petri dish to treat seedlings with GR24 5DO, the other one for the untreated control condition. Separate the seeds into treatment groups already during sowing to reduce contamination risks and facilitate handling.
  b. Moisten the filter paper slightly with sterile autoclaved deionized water.
  c. Pour all imbibed seeds into the Petri plate and distribute the seeds evenly on the filter paper using sterilized tweezers.
  d. Pipette out the excess water accumulated inside the Petri plate.
    ***Note:*** Ensure the filter paper is evenly moist but not oversaturated.
  e. Close the Petri dish and seal it with sterile micropore tape to prevent contamination (Figure 1F).
  f. Transfer the sealed dish to 4°C and incubate in the dark for 6 weeks for stratification.
    ***Note:*** During stratification at 4°C, inspect the plates weekly to ensure the filter paper remains moist (see troubleshooting step below). If necessary, carefully open the plates inside a laminar airflow cabinet and add a small volume of sterile autoclaved water to maintain consistent moisture levels.
11. Sow *C. affinis* seeds on F-medium for plant-plant interaction assays.
  a. Prepare flat F-medium plates inside a sterile laminar airflow cabinet, ensuring the medium is fully solidified before sowing seeds.
  b. Transfer seeds to a sterile Petri plate by pouring the imbibed *C. affinis* seeds into a sterile Petri dish for easy handling (Figure 1C).
  c. Place seeds on medium.
  d. Using sterilized tweezers, pick up individual seeds and carefully place them on the surface of the F-medium.
  e. Align them in rows, ensuring even spacing (Figure 1D).
  f. Seal the plates using sterile micropore tape to maintain sterility.
  g. Transfer the plates to 4°C for 6–10 weeks of dark stratification with continuous monitoring after 6 weeks.
    ***Note:*** In F-medium, seeds of *C. affinis* start germinating as early as 25 days (Figure 2A) in the dark during stratification. While a minimum of 6 weeks of stratification leads to cotyledon production and short root emergence, the roots at this stage are too fragile for transfer onto slanted plates. Therefore, after 6 weeks, we continued to monitor the seedlings until roots reached 1–2 cm in length and cotyledons fully emerged. This extended period resulted in a total stratification time of 10 weeks, ensuring seedlings were sufficiently developed for easier handling and transfer (Figure 2A). By the end of 10 weeks, nearly 100% of seedlings displayed cotyledons and elongated roots. It is essential to maintain sterility throughout the process by performing all steps inside the laminar airflow cabinet and handling seeds with ethanol-wiped or flame-sterilized tweezers to prevent contamination.

### Hormone treatment and plant-plant interaction assay

**Timing: 3–7 days (seedling growth) + 1 h (microscopy)**

**Figure 3:**
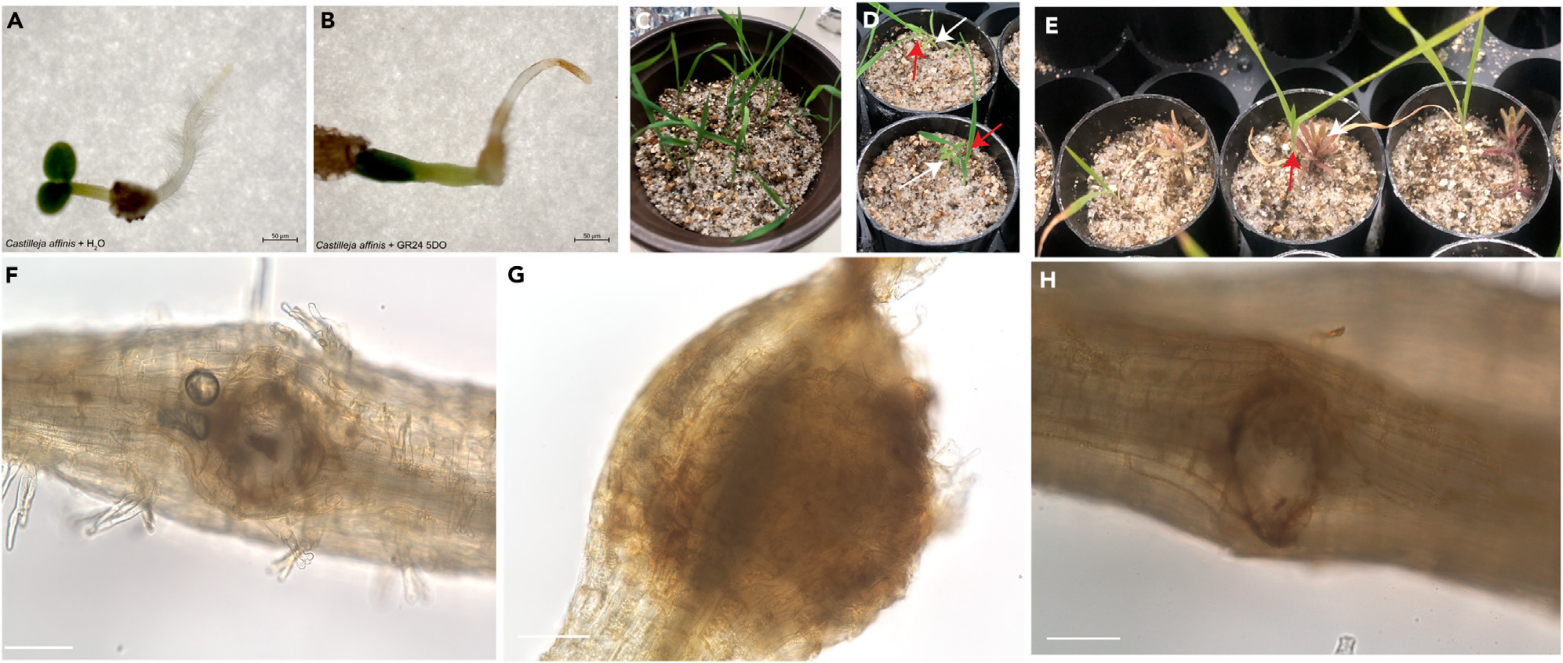
Strigolactone treatment in *Castilleja affinis* and plant-plant interaction assay (A and B) *C. affinis* seedlings grown under (A) control conditions (H_2_O) and (B) 1 μM GR24 5DO. Haustorium-like structures are observed in GR24 5DO-treated seedlings. (C) *Brachypodium distachyon* seedlings germinated in sand-vermiculite mix before co-cultivation with *C. affinis*. (D and E) *C. affinis* seedlings co-cultivated with *B. distachyon*. (D) 1^st^ day and (E) 18^th^ day. White arrows indicate *C. affinis* seedlings, and red arrows indicate *B. distachyon* hosts. (F–H) Microscopy images of haustorium structures in *C. affinis* roots. Scale bar: 100 nm. (A) and (B) are adopted from Figure 1 in Bürger et al.^1^

***Note:*** Within 3–4 days of removing the plates from cold stratification and application of either H_2_O or GR24 5DO, there should be visible seedling growth on both plates. Seedlings on the water-treated plates should show clear development of a root system with root hairs. Seedlings in the GR24 5DO-treated plates should be hairless and devoid of lateral roots but feature a reddish/brownish terminal structure. This timeframe can be species-specific, and results might take up to 7 days to fully develop.

12. Carefully transfer the Petri dishes with the *Castilleja* seeds to a laminar airflow cabinet to maintain sterility.
13. Gently remove the micropore tape from the Petri dish, ensuring minimal disturbance to the stratified seedlings.
14. Set up two experimental conditions:
  a. GR24 5DO-treated plate: Apply 500 μL of 1 μM GR24 5DO working solution evenly onto the filter paper.
  b. Control plate: Apply 500 μL of sterile autoclaved water, containing 0.01% DMSO.
15. Ensure that both plates have a consistent moisture level and remove any excess liquid using a sterile pipette if necessary.
16. Reseal the Petri dishes with sterile micropore tape and transfer them to a growth chamber at room temperature under a 16-h light/8-h dark cycle for germination.
17. Monitor and document root development (Figure 3A) and haustorium-like structure formation over the next 5 days (Figure 3B).

18. Conduct Microscopy.
  ***Note:*** In our assay, we observed *C. affinis* seedlings directly on Whatman filter paper without fixation to preserve their native structure.
  a. Capture high resolution images using a Zeiss Axio Zoom V16 (or microscope of your choice) microscope in bright-field mode.
    ***Note:*** Adjust image brightness and contrast to enhance clarity, if necessary.

### Plant-plant interaction assay

**Timing: 8 days (host seedling preparation) + 30–45 days (co-cultivation) + 2 days (harvest and microcopy)**

19. Prepare host plant seedlings.
  a. *Brachypodium distachyon* seeds were sterilized using the same protocol as *Castilleja* seeds (Step 6).
  b. Stratify seeds for 4 days at 4°C in the dark to ensure uniform germination.
  c. Stratified seeds were sown in 4-inch pots filled with a 2:1 mixture of Quikrete play sand and vermiculite (Figure 3C).
  d. Keep pots moist at room temperature under a 16-h light/8-h dark cycle for seed germination.

***Note:*** For optimal co-cultivation with *Castilleja*, transfer seedlings when their shoots are 1.5–2 cm long, although larger seedlings (up to 5 cm, which seedlings reached after ∼12 days, Figure 3C) can be used successfully.

***Note:*** Instead of germinating in a sand-vermiculite mix, *Brachypodium* seedlings can also be germinated on Whatman filter paper following the same procedure as *Castilleja* seeds. Using this method, seedlings typically germinate within 3–4 days and can then be transferred to potting substrate for further growth.

20. Prepare growth cone-tainers and substrate.
  a. Prepare and label 20.5 cm-long cone-tainers for planting.
  b. Fill the cone-tainers with a sterile mixture of Quikrete play sand and vermiculite in a 2:1 ratio, excluding approximately 1.5 cm from the top to allow space for watering of the plants.

**CRITICAL:** The sand and vermiculite mixture should be washed and autoclaved before use to prevent microbial contamination.

21. Transplant and co-cultivate.
  a. Moisten the sterile sand-vermiculite mixture with DI water before transplanting to provide an optimal rooting environment.
  b. Using a sterile 1 mL pipette tip, create a small depression (∼1.5 cm deep) in the substrate.
  c. Carefully transplant one *Castilleja* seedling alongside one *Brachypodium* seedling at the same time to facilitate root interaction (Figure 3D).
    ***Note:****Castilleja* seedlings should be handled gently to avoid mechanical damage.
  d. Gently cover the roots with the sand-vermiculite mix while avoiding damage to root tips.
22. Water and maintain plant growth.
  a. Transfer the cone-tainers to a growth chamber set to:
    i. 16-h light (25°C)/8-h dark (22°C) cycle.
    ii. 40% relative humidity.
  b. Apply 15 mL of ½ Hoagland’s solution with 20 μM phosphate twice a week (every 4 days) to support plant nutrition and root interactions.

***Note:*** Tailor the experimental approach to the specific research objective. In our method, we used low phosphate (20 μM KH_2_PO_4_) nutrient solution; however, we recommend that researchers adjust the nutrient composition based on their experimental aims. For example, different nutrient compositions may be tested to study their effects on plant-plant interactions. Nutrient composition can also be varied if the system is co-inoculated with microbes to examine tripartite interactions.

***Note:*** After 18 days of watering, we observed that the host plant leaves began to exhibit a pale coloration, while *C. affinis* developed a pinkish-green appearance. This may be a response to low phosphate conditions and may vary depending on the growth environment (Figure 3E).

23. Harvest the plants.
  ***Note:*** In this protocol, *Castilleja* and *Brachypodium* host plants were harvested after 18 days of co-cultivation; however, However, depending on research goals, longer co-cultivation times should be considered.
  a. Label one 15 mL Falcon tubes for each sample to be harvested.
    ***Note:*** It is recommended to harvest roots near a sink to facilitate rinsing of samples and clean-up.
  b. Gently squeeze the cone-tainers with your hand to loosen the sand-vermiculite mixture.
  c. Tap gently on a collection tray to dislodge excess sand.
  d. Carefully remove the plants from the cone-tainers and separate them from the remaining substrate.
  e. Transfer the plants into a 1-L beaker filled with sterile water, ensuring roots are submerged.
  f. Gently shake the roots in the water to remove any attached sand.
    ***Note:*** Handle plants gently during sand removal to avoid root damage. Some vermiculite may remain attached to the roots, which is acceptable for visualizing haustorium structures but should be carefully removed depending on the application.
24. Visualize and microscope the result.
  a. Perform an initial assessment of haustorium structures in the whole root using a stereomicroscope.
  b. Cut roots into small (approx. 1–2 cm long) sections using a blade and mount them on glass slides for imaging with a Keyence BZ-X810 microscope under a 20x lens. This allows for visualization of the haustorias' characteristic bell-shaped structure, which initially appears as a spherical swelling accompanied by localized root hairs (Figures 3F–3H). The haustorium consists of several distinct zones: a central core of collenchyma tissue, surrounded by parenchymatous regions, and vascular tissue that connects the parasite to its host.^3^

***Note:*** We recommend clearing roots in 20% KOH for 3–4 days at 65°C, followed by rinsing 3–5 times with distilled water (see troubleshooting step below), before mounting samples on slides to enhance the visualization of cellular structures and improve imaging clarity. It is important to note that in this study, haustorium formation was not quantified or systematically monitored, as comparative experimental conditions were not included. Instead, the primary goal was to develop a method for establishing haustorium structures in a living host-parasite interaction under controlled lab conditions. Additionally, we suggest using the Leica M205 FA or a similar stereomicroscope to quantify the total number of haustorium structures in a single root system prior to sectioning, as it provides a practical tool for conducting quantitative analyses. The approach described here provides a foundation for future studies aiming to analyze host-parasite dynamics in greater detail.

## Expected outcomes

A successful implementation of this protocol results in efficient germination of *Castilleja* seeds and development of haustorium structures for plant-plant interaction studies. When following the stratification and germination procedures outlined, researchers can expect approximately 80–100% germination rates after 10 weeks, with seedlings developing visible cotyledons and 1–2 cm roots (Figure 2A). Following GR24 5DO treatment, haustorium-like structures should be observable within 3–7 days as distinct reddish/brownish terminal structures (Figure 3B). In plant-plant interaction assays with *Brachypodium distachyon*, functional haustoria connecting the parasite to the host root system should be visible after 18 days of co-cultivation, appearing as characteristic bell-shaped structures with localized root hairs (Figures 3F–3H). These structures demonstrate successful establishment of the parasitic relationship under controlled laboratory conditions.

## Limitations

This protocol provides methods for studying *Castilleja* parasitism and haustorium formation under controlled conditions. As with any model system, there are limitations that should be considered when interpreting results. In this protocol, *Castilleja* seedlings are grown under controlled humidity, temperature, and nutrient conditions in cone-tainers filled with a sterile sand-vermiculite mixture. While this setup allows for close monitoring of haustorium development, it does not fully replicate natural soil environments, where interactions with microbial communities, organic matter, and fluctuating nutrient availability may influence plant development and parasitism efficiency. Additionally, while chemical induction of haustorium-like structures using GR24 5DO provides a valuable tool for studying this developmental process, these structures may not fully replicate the complexity of host-induced haustoria in terms of their development, structure, or function, given the multitude of different strigolactone molecules in the rhizosphere. Haustoria may detach from host roots during harvesting, making it difficult to accurately assess attachment efficiency. While plants were harvested at 18 days, haustorium formation is an ongoing process, and optimal observations may require extending the experiment to 30–45 days. Despite these challenges, this method provides an effective system for investigating *Castilleja* parasitism under controlled conditions.

## Troubleshooting

### Problem 1: Low seed germination rate

During seed stratification (Steps 10-15), seeds may not undergo sufficient chilling on Whatman filter or F-medium. Dried out filter paper may also lead to poor germination.

### Potential solution

Ensure seeds are stratified for at least 6 weeks at 4°C in the dark which should be sufficient time for further germinating the seedlings in the light. If germination is still low, extend stratification up to 10 weeks. If seeds are germinating on Whatman filter paper, plates should be checked on a weekly basis and sterile autoclaved water should be added as needed to maintain moisture levels. Seed germination efficiency also depends on the seed selection (Steps 1-3).

### Problem 2: Contamination

Contamination can arise from improper sterile technique (Steps 10-25), including the use of unclean tweezers, gloves, or exposure to non-sterile conditions during seed handling and plating.

### Potential solution

Maintain strict sterile conditions by using autoclaved tools, wearing gloves, and working in a laminar flow cabinet. Discard any contaminated plates to prevent the spread of unwanted microbes.

### Problem 3: Haustoria detachment during harvesting

Haustoria may become detached from host roots if the root systems are handled without care or are exposed to excessive rinsing during sample collection (Step 46-50). This can affect experimental results and prevent observation of haustoria attachments.

### Potential solution

To minimize haustoria detachment, carefully extract plants from the substrate while avoiding excessive force. Minimize shaking during root washing, as vigorous movement can dislodge haustoria. Using soft forceps to gently handle the roots and employing a slow, controlled rinsing technique with minimal water pressure can help preserve haustoria attachment.

### Problem 4: Carefully harvested but haustoria attachment not observed in microscope

#### Potential cause

If haustoria are not visible under the microscope, it could be due to an insufficient co-culture growth period, preventing their full development. Additionally, experimental conditions (e.g., sub-optimal nutrient conditions) may not support haustorium formation, affecting successful host-parasite interaction.

### Potential solution

To improve haustorium formation, extend the co-culture growth period with the host plant to allow more time for development. Additionally, optimize the nutrient composition of the growth medium to create favorable conditions for symbiotic interaction.

### Problem 5: Root clearing

Root cells may be difficult to visualize due to tissue opacity, making it challenging to observe structures such as haustoria.

### Potential solution

To enhance tissue transparency, use clearing agents such as potassium hydroxide (KOH) to clear tissue layers before microscopy. Adjust the concentration and incubation time based on root thickness to achieve optimal clarity while preserving structural integrity.

### Problem 6: No or poor haustorium development during GR24 5DO treatment

Poor haustorium development may be due to the degradation of GR24 5DO (Step 28). If the stock solution degrades or precipitates, its effectiveness in triggering haustorium development can be significantly reduced.

### Potential solution

Check the GR24 5DO stock solution for signs of degradation, such as precipitation or discoloration. If precipitation is observed, discard the stock and prepare a fresh GR24 5DO solution. To ensure consistency in haustorium induction, always prepare a fresh 1 mM GR24 5DO working solution before each experiment. Store stock solutions properly, following recommended light, temperature, and solvent conditions to maintain stability.

## Resource availability

### Lead contact

Further information and requests for resources and reagents should be directed to and will be fulfilled by the lead contact, Marco Bürger (mburger@salk.edu).

### Technical contact

Technical questions on executing this protocol should be directed to and will be answered by the technical contact, Marco Bürger (mburger@salk.edu).

### Materials availability

This study did not generate new unique reagents.

### Data and code availability

This study did not generate new data or code.

## Acknowledgments

We extend our gratitude to J. Mark Egger (Herbarium, Burke Museum of Natural History and Culture, University of Washington) for help with *Castilleja* species identification. We thank Yerisf C. Torres Ascurra (Salk Institute for Biological Studies) for useful insights and discussion. This project was funded by start-up support from the Salk Institute for Biological Studies and the Hess Foundation to L.M.M. This study has been supported by the National Institutes of Health (NIH) grant R35 GM122604 to J.C. J.C. is an investigator of the Howard Hughes Medical Institute. The graphical abstract was created using Biorender.com.

## Author contributions

S.B. and M.B. developed and optimized the protocol. S.B. drafted the initial protocol and prepared the figures, which were further updated by M.B. with input from L.M.M. S.B., L.M.M., and M.B. edited the protocol. L.M.M. and M.B. supervised the project. Funding acquisition was supported by L.M.M. and J.C.

## Declaration of interests

The authors declare no competing interests.
